# Coproduction in Social Prescribing Initiatives: Protocol for a Scoping Review

**DOI:** 10.2196/57062

**Published:** 2024-10-17

**Authors:** Madeline Dougherty, Tamara Tompkins, Elaine Zibrowski, Jesse Cram, Maureen C Ashe, Le-Tien Bhaskar, Kiffer George Card, Christina Godfrey, Paul Hebert, Ron Lacombe, Caitlin Muhl, Kate Mulligan, Gillian Mulvale, Michelle L A Nelson, Myrna Norman, Bobbi Symes, Gary Teare, Vivian Welch, Anita Kothari

**Affiliations:** 1 Faculty of Health and Rehabilitation Sciences Western University London, ON Canada; 2 Faculty of Medicine University of British Columbia Vancouver, BC Canada; 3 Department of Health Research, Evidence, and Impact McMaster University Hamilton, ON Canada; 4 Faculty of Health Sciences Simon Fraser University Burnaby, BC Canada; 5 School of Nursing Collaboration for Health Care Quality: A JBI Centre of Excellence Queen's University Kingston, ON Canada; 6 School of Epidemiology and Public Health University of Ottawa Ottawa, ON Canada; 7 Patient Advisory Counsel Canadian Institute for Social Prescribing Toronto, ON Canada; 8 Faculty of Health Sciences Queen's University Kingston, ON Canada; 9 Institute of Dalla Lana School of Public Health University of Toronto Toronto, ON Canada; 10 Lunenfeld-Tanenbaum Research Institute Sinai Health System Toronto, ON Canada; 11 United Way British Columbia Burnaby, BC Canada; 12 Alberta Health Systems Edmonton, AB Canada; 13 Bruyère Research Institute Ottawa, ON Canada

**Keywords:** social prescribing, coproduction, codevelopment, policy, social prescription, nonmedical need, social need, clinical setting, community programs, policy, health care system, pilot-tested, user involvement, health education

## Abstract

**Background:**

Social prescribing (SP) takes a holistic approach to health by linking clients from clinical settings to community programs to address their nonmedical needs. The emerging evidence base for SP demonstrates variability in the design and implementation of different SP initiatives. To effectively address these needs, coproduction among clients, communities, stakeholders, and policy makers is important for tailoring SP initiatives for optimal uptake.

**Objective:**

This study aims to explore the role of coproduction in SP initiatives. The research question is as follows: How and for what purpose has coproduction been incorporated across a range of SP initiatives for different clients?

**Methods:**

A review of international literature will be conducted following the JBI guidelines for scoping reviews. We will search multiple databases including Scopus, MEDLINE, and the PAIS Index, as well as gray literature, from 2000 to 2023. The primary studies included will describe a nonmedical need for clients, a nonmedical SP program or initiative, coproduction of the SP program, and any follow-up. Review articles and commentaries will be excluded. Titles, abstracts, and full-text articles will be screened, and data will be extracted by at least 2 research team members using Covidence and a pilot-tested extraction template. Clients with lived experience will also participate in the research process. Findings will be descriptively summarized and thematically synthesized to answer the research question.

**Results:**

The project was funded in 2023, and the results are expected to be submitted for publication in early 2025.

**Conclusions:**

Descriptions of what coproduction is meant to accomplish may differ from theoretical aspirations. Continued understanding of how coproduction has been designed and executed across varied international SP models is important for framing engagement in practice for future SP arrangements and their evaluation. We anticipate this review will guide clients, communities, stakeholders, and policy makers in further developing SP practice within health care systems.

**Trial Registration:**

Open Science Framework Registries B8U4Z; https://osf.io/b8u4z

**International Registered Report Identifier (IRRID):**

DERR1-10.2196/57062

## Introduction

### Background

The World Health Organization (WHO) defines social determinants of health (SDOH) as the conditions in which people are “born, grow up, live, work and age, and the systems put in place to deal with illness” [1]. The WHO estimates that 30% to 55% of individual health outcomes are accounted for by SDOH, and many outside factors affecting SDOH can strongly influence health equity, both positively and negatively [2]. SDOH include education, food security, housing, social inclusivity, and nondiscrimination [1]. Although health care providers are aware of and acknowledge the influence of SDOH on overall health and well-being, education and resources supporting their abilities to effectively address these needs are often limited [3-6].

The United Kingdom was among the first countries to develop a national long-term plan in addressing health inequities related to SDOH [7]. They have had success in doing this through their National Health Service *Long Term Plan* by linking patients from primary care to nonmedical resources and support through their communities, termed social prescribing (SP). SP allows health care providers to enable patients to connect to personalized, nonclinical supports and services in their community. It also empowers patients to have an active role in coproducing, along with their health care team, individualized prescriptions for improving their overall health and well-being through existing social resources. SP is a first step in helping to decrease the effect of social inequities [7-11]. Other countries have since implemented and invested funding and resources into SP initiatives [7,12]. Although the terminology differs worldwide, there are currently 17 countries around the world that have developed and implemented SP initiatives. In addition, an international SP network was formed in 2015 [7].

Research has shown that SP can improve social connectedness and belonging by helping people develop social support networks within individual communities, resulting in improved self-confidence, self-esteem, and empowerment [13,14]. SP enables individuals to actively address challenging, personal, nonmedical needs, which can lead to improvements in long-term health outcomes, such as better management of mental health conditions like anxiety and depression [9,10,15,16].

Although worldwide evidence on SP initiatives is promising, the literature remains inconsistent, with most studies consisting of small-scale evaluations with poor study design and reporting [9]. Since SP was first described in the 1990s, the discourse around it has continuously evolved, but there is still no universal service model [7]. A service model would describe how health care providers, social workers, community organizations, and other stakeholders collaborate to connect individuals with nonclinical supports and services. Previous literature has described a need for consistent frameworks across SP programs and evaluations, although varying definitions and program structures present challenges. On the individual level, SP initiatives can also vary in terms of client population, the activities and stakeholders involved, duration of support provided to clients, and the resources required to address the intended social needs [7,13,17]. Understanding the various facets underpinning SP initiatives is important for achieving collective scalability, widespread adoption, and sustainable impact at both the individual and health systems level, ultimately aiming to mitigate health inequities.

### What This Scoping Review Will Contribute

Emerging evidence from 1 UK region identified public-service user involvement as a key factor in the successful delivery of a SP scheme [17]. Clients’ adherence to the program was maximized when they co-designed their social prescriptions through detailed assessment of their needs and preferences while considering potential access barriers. The involvement of local communities as coproducers and designers was also noted to be important in the development of place-based services within the region [17]. A recent systematic review of SP interventions that utilized a coproduction approach to improve well-being identified 8 relevant articles [9]. The review found that such engagement resulted in effective and efficient SP interventions and improved well-being outcomes [9].

Coproduction involves working with stakeholders to implement a solution that has been agreed upon for a specific problem or established need. It emphasizes the allocation of resources and assets within these set boundaries to achieve improved outcomes [18]. Continued understanding of how coproduction has been designed and executed across varied international SP models is important for framing engagement in practice for future SP arrangements and their evaluation. As previous research and systematic reviews have indicated, there is a need to better understand the mechanisms, or “black box,” behind coproduction processes across various SP models [9].

The objective of this scoping review is to explore the role of coproduction in SP initiatives. Empirically based descriptions of what coproduction is meant to accomplish may differ from theoretical aspirations. The findings from this review could provide further guidance for evaluating SP initiatives, ultimately leading to improved services, better client outcomes, and a reduction in health inequities through the global development of SP programs [19].

## Methods

### Design

This scoping review will adhere to the guidance provided by JBI and follow updated recommendations for this methodology [19,20]. The development and quality of this protocol were informed by the PRISMA-P (Preferred Reporting Items for Systematic Review and Meta-Analysis Protocols) guidelines [21]. The review’s conduct and reporting will align with the PRISMA-ScR (Preferred Reporting Items for Systematic Reviews and Meta-Analyses Extension for Scoping Reviews) checklist as recommended by JBI [22]. Both the PRISMA-P and PRISMA-ScR provide a framework that ensures rigorous reporting of the review process and results. This scoping review will involve the following prescribed processes: (1) creating the research question; (2) developing the search strategy; (3) screening, selecting, and appraising studies; (4) extracting data; and (5) analyzing and reporting data. The protocol has been registered with the Open Science Framework Registries (B8U4Z) [23].

### Creating the Research Question

The research question and overall approach were developed in collaboration with the larger research team, which includes members with lived experience and expertise in SP and coproduction. The approach also incorporates scoping review methodology and draws on the Population, Intervention, Comparison, Outcomes framework as outlined in PRISMA-P, although no comparison group was included.

The scoping review research question is as follows: How and for what purpose has coproduction been incorporated (outcome) across a range of SP initiatives (intervention) for different clients (population)? The term “client” refers to individuals and communities.

### Search Strategy Development

The search strategy will be refined in collaboration with the research team, including those with professional expertise and experience in developing search strategies for a scoping review. The search will include OVID MEDLINE, Scopus, and the PAIS Index using a variety of keywords developed from concepts related to coproduction and SP. A pilot search on Scopus was conducted to aid the development of a comprehensive search strategy, including subject headings and search terms. The final strategy for identifying published literature will include database-specific subject headings and keywords. Additionally, we will review the reference lists of included studies and relevant review articles to identify potentially relevant studies. While we considered hand-searching, we determined it unnecessary since scholars in the emerging field of SP have not yet published extensively in key research journals. The search will not be limited by language or study design but will focus on literature from 2000 to 2023, as preliminary research indicated that the concept of SP is relatively new, with reports of SP programs appearing in the early 2000s [22,24]. Multimedia Appendix 1 presents the search string developed in consultation with an information specialist.

### Evidence Screening, Selection, and Appraisal

We will include studies describing an SP program or initiative incorporating coproduction in its approach. For the purposes of this scoping review, SP is defined as “a means for trusted individuals in clinical and community settings to identify that a person has non-medical, health-related social needs and to subsequently connect them to non-clinical supports and services within the community by co-producing a social prescription—a non-medical prescription, to improve health and well-being and to strengthen community connections” (p. 9) [3]. The term “trusted individual” refers to service providers who may not be health care professionals but are trusted within the community by those they serve; these individuals, also known as identifiers, are considered part of SP programs [3]. The required conditions for SP outlined by Muhl et al [3] were rigorously developed with input from the international SP community, including researchers and other stakeholders, using a Delphi approach. These conditions are as follows: SP is “a holistic, person-centred, and community-based approach to health and well-being that satisfies Condition 1 and either Condition 2 or Conditions 3 and 4:

- Condition 1: Identifier identifies that person has non-medical, health-related social needs (e.g., issues with housing, food, employment, income, social support)
- Condition 2: Identifier connects person to non-clinical supports and services within the community by co-producing a non-medical prescription
- Condition 3: Identifier refers person to connector
- Condition 4: Connector connects person to non-clinical supports and services within the community by co-producing a non-medical prescription” [3].

A connecter, also part of the SP program, is responsible for linking the person to community resources. This role may be filled by the same person as the identifier or by someone else. The connector is sometimes referred to as the link worker. Muhl et al [3] also developed a Common Understanding of Social Prescribing Conceptual Framework, which outlines that the connector follows up with the person and reports back to the identifier.

Based on our research question and the specified conditions, we will select articles that (1) were published between 2000 and 2023; (2) are peer-reviewed primary research, including qualitative, quantitative, or mixed methodology studies; (3) include information on the client and/or community in the coproduction of the SP initiative (population); (4) describe an SP initiative, including the identification of a nonmedical needs for the client (intervention); (5) describe an SP initiative with follow-up, such as feedback, to the SP identifier or connector regarding the client (intervention); and (6) describe the client and/or community engagement process in the coproduction of the SP initiative (outcome). We will also search for gray literature, including resources disseminated by international SP initiatives, which may encompass local program evaluations and/or review articles commissioned by the initiatives. We will examine the reference lists of these resources for additional relevant studies.

Results from the database and gray literature searches will be exported to and managed through Covidence (Veritas Health Innovation), a web-based collaboration platform that streamlines the production of systematic and other literature reviews [25]. After removing duplicates, the screening process will involve 2 rounds: an initial screening of titles and abstracts, followed by a full-text review. A pilot test with randomly selected articles will be conducted by at least 4 research team members to clarify the inclusion/exclusion criteria and ensure consistent screening. Through repeated iterations and discussions, we will reach a common understanding of the study concepts. Subsequently, 2 raters will review all articles for eligibility. Eligible articles will proceed to the second round of screening. Any conflicts in determining article eligibility will be resolved by a third reviewer. The second round will involve a detailed, full-text screening against our eligibility criteria. Reviewers will document the reason for excluding articles based on these criteria [25]. We will not conduct a quality or risk assessment of the articles, as this is not typically part of the scoping review methodology [22].

### Data Extraction

The team will use the *JBI Manual* data extraction guidance to develop our data extraction template. We anticipate recording the following characteristics from each article: (1) title, (2) author, (3) year of publication, (4) country involved, (5) type of literature, (6) purpose/goal/aim of the article, (7) study methodology, and (8) health care setting. In addition to these metadata characteristics, we will extract information about the SP initiative, including the clients involved, their nonmedical needs, the role of the connector or link worker, the nature of the social prescription, and follow-up with the initiator. We will also collect data on the coproduction process at both the community and client levels to understand the reasons for and methods of engagement.

A pilot extraction process will be conducted to refine the template. Three team members will participate in the extraction process. Each article will be initially extracted by 1 researcher and subsequently reviewed by others to ensure consistency. The team will meet regularly to discuss their extractions and ensure alignment.

### Data Analysis and Reporting

The metadata of the included studies will be summarized with descriptive statistics. Qualitative content analysis will be employed to synthesize data about the SP initiative and how coproduction was utilized [26]. The reporting of this scoping review will follow the PRISMA-ScR checklist recommended by JBI [22].

### Client Involvement

Two coresearchers with lived experience, who are members of Canadian Institute for Social Prescribing Patient Advisory Committee, will be involved in the scoping review process. They are expected to contribute to 1 or more of the following activities: discussing and reflecting on study findings during research team meetings, supporting the development of our gray literature strategy, and assisting in the creation of a lay summary. The coresearchers will be offered an honorarium consistent with the Canadian Institute for Social Prescribing’s guidelines.

### Ethical Considerations

This scoping review involves a secondary analysis of primary research studies and gray literature on coproduction in SP initiatives; therefore, institutional research ethics approval is not required.

## Results

The project was funded in 2023, and the results are expected to be submitted for publication in early 2025.

## Discussion

### Expected Findings

This scoping review will serve as a foundation for a larger research study aimed at further evaluating and understanding the importance of coproduction and SP as they become increasingly common practices across Canada. Both traditional and nontraditional dissemination strategies will be employed to broaden the reach of the review’s findings.

Coproduction approaches, which can lead to more engaging, usable, relevant, and effective programs, are gaining momentum [27,28]. Through meaningful and reciprocal engagement, coproduction can support shared responsibility and ownership of programs and interventions [27]. There is mounting evidence that using coproduction to design SP initiatives is beneficial, but there is limited consistency in understanding how this has been accomplished. The proposed scoping review will synthesize the evidence on the role of the client or community in coproducing SP initiatives. This review will identify where current practices related to coproduction for SP diverge and converge.

### Limitations

Challenges are anticipated in the search process due to inconsistencies in terminology related to SP. For example, articles describing SP initiatives from different countries may use varied language. While our research team, including experts in the field, will strive to compile a comprehensive list of search terms to encompass all iterations of SP in the literature, it is possible that some SP terms may be overlooked and relevant articles may be missed.

### Conclusions

The existing literature lacks a comprehensive understanding of coproduction in SP initiatives, which is important for leveraging community and client expertise in developing this integrated care model. This scoping review aims to address the knowledge gap regarding the role of coproduction in SP initiatives. We anticipate the findings will guide clients, communities, stakeholders, and policy makers in further developing SP practices and coproduction options within health care systems.

## Appendix

Multimedia Appendix 1 Search strategy for OVID MEDLINE.

## Abbreviations

PRISMA-P: Preferred Reporting Items for Systematic Review and Meta-Analysis Protocols
PRISMA-ScR: Preferred Reporting Items for Systematic Reviews and Meta-Analyses Extension for Scoping Reviews
SDOH: social determinants of health
SP: social prescribing
WHO: World Health Organization

## References

[1] (2008). Closing the gap in a generation: health equity through action on the social determinants of health - final report of the Commission on Social Determinants of Health. World Health Organization.

[2] Social determinants of health. World Health Organization.

[3] Muhl C, Mulligan K, Bayoumi I, Ashcroft R, Godfrey C (2023). Establishing internationally accepted conceptual and operational definitions of social prescribing through expert consensus: a Delphi study. BMJ Open.

[4] Andermann A, CLEAR Collaboration (2016). Taking action on the social determinants of health in clinical practice: a framework for health professionals. CMAJ.

[5] Kung A, Cheung T, Knox M, Willard-Grace R, Halpern J, Olayiwola JN, Gottlieb L (2019). Capacity to address social needs affects primary care clinician burnout. Ann Fam Med.

[6] Pantell MS, de Marchis E, Bueno A, Gottlieb LM (2019). Practice capacity to address patients' social needs and physician satisfaction and perceived quality of care. Ann Fam Med.

[7] Morse DF, Sandhu S, Mulligan K, Tierney S, Polley M, Chiva Giurca B, Slade S, Dias S, Mahtani KR, Wells L, Wang H, Zhao Bo, de Figueiredo CEM, Meijs JJ, Nam HK, Lee KH, Wallace C, Elliott M, Mendive JM, Robinson D, Palo M, Herrmann W, Østergaard Nielsen Rasmus, Husk K (2022). Global developments in social prescribing. BMJ Glob Health.

[8] Araki K, Takahashi Y, Okada H, Nakayama T (2022). Social prescribing from the patient's perspective: a literature review. J Gen Fam Med.

[9] Thomas G, Lynch M, Spencer LH (2021). A systematic review to examine the evidence in developing social prescribing interventions that apply a co-productive, co-designed approach to improve well-being outcomes in a community setting. Int J Environ Res Public Health.

[10] Ebrahimoghli R, Pezeshki MZ, Farajzadeh P, Arab-Zozani M, Mehrtak M, Alizadeh M (2023). Factors influencing social prescribing initiatives: a systematic review of qualitative evidence. Perspect Public Health.

[11] Polley MJ, Fleming J, Anfilogoff T, Carpenter A (2017). Making sense of social prescribing. University of Westminster.

[12] Costa A, Sousa CJ, Seabra PRC, Virgolino A, Santos O, Lopes J, Henriques A, Nogueira P, Alarcão V (2021). Effectiveness of social prescribing programs in the primary health-care context: a systematic literature review. Sustainability.

[13] Mulligan K, Hsiung S, Bloch G, Park G, Richter A, Stebbins L, Talat S (2023). Social prescribing in Canada: a tool for integrating health and social care for underserved communities. Healthc Q.

[14] Canadian Institute for Social Prescribing.

[15] Grover S, Sandhu P, Nijjar G, Percival A, Chudyk A, Liang J, McArthur C, Miller W, Mortenson W, Mulligan K, Newton C, Park G, Pitman B, Rush K, Sakakibara B, Petrella R, Ashe M (2023). Older adults and social prescribing experience, outcomes, and processes: a meta-aggregation systematic review. Public Health.

[16] Cooper M, Avery L, Scott J, Ashley K, Jordan C, Errington L, Flynn D (2022). Effectiveness and active ingredients of social prescribing interventions targeting mental health: a systematic review. BMJ Open.

[17] Hassan SM, Ring A, Goodall M, Abba K, Gabbay M, van Ginneken N (2023). Social prescribing practices and learning across the North West Coast region: essential elements and key challenges to implementing effective and sustainable social prescribing services. BMC Health Serv Res.

[18] Vargas C, Whelan J, Brimblecombe J, Allender S (2022). Co-creation, co-design, co-production for public health - a perspective on definition and distinctions. Public Health Res Pract.

[19] Peters MDJ, Godfrey C, McInerney P, Munn Z, Tricco AC, Khalil H, Aromataris E, Lockwood C, Porritt K, Pilla B, Jordan Z (2020). Scoping reviews. JBI Manual for Evidence Synthesis.

[20] Khalil H, Peters MD, Tricco AC, Pollock D, Alexander L, McInerney P, Godfrey CM, Munn Z (2021). Conducting high quality scoping reviews-challenges and solutions. J Clin Epidemiol.

[21] Moher D, Shamseer L, Clarke M, Ghersi D, Liberati A, Petticrew M, Shekelle P, Stewart L, PRISMA-P Group (2015). Preferred reporting items for systematic review and meta-analysis protocols (PRISMA-P) 2015 statement. Syst Rev.

[22] Tricco AC, Lillie E, Zarin W, O'Brien KK, Colquhoun H, Levac D, Moher D, Peters MDJ, Horsley T, Weeks L, Hempel S, Akl E, Chang C, McGowan J, Stewart L, Hartling L, Aldcroft A, Wilson M, Garritty C, Lewin S, Godfrey CM, Macdonald MT, Langlois EV, Soares-Weiser K, Moriarty J, Clifford T, Tunçalp Ö, Straus SE (2018). PRISMA extension for scoping reviews (PRISMA-ScR): checklist and explanation. Ann Intern Med.

[23] Exploring the incorporation of co-production and policy in social prescribing initiatives. Open Science Framework Registries.

[24] Friedli L, Watson S (2004). Social prescribing for mental health.

[25] Covidence systematic review software. Veritas Health Innovation.

[26] Stemler S (2000). An overview of content analysis. Practical Assessment, Research, and Evaluation.

[27] Slattery P, Saeri AK, Bragge P (2020). Research co-design in health: a rapid overview of reviews. Health Res Policy Syst.

[28] Grindell C, Coates E, Croot L, O'Cathain A (2022). The use of co-production, co-design and co-creation to mobilise knowledge in the management of health conditions: a systematic review. BMC Health Serv Res.

