# Supplementary material for: Coproduction in Social Prescribing Initiatives: Protocol for a Scoping Review
Source: JMIR Res Protoc. 2024 Oct 17;13:e57062. doi: 10.2196/57062 (PMC11528170; doi:10.2196/57062)
Supplement: Multimedia Appendix 1 [file resprot_v13i1e57062_app1.docx]

Multimedia Appendix 1 – Search strategy for OVID MEDLINE

**July 02 2023 OVID MEDLINE

(codesign or "co-design" or coproduction or "co-production" or cocreat* or "co-creat*" or collaborat* or engag* or involve* or codevelop* or "co-develop*" or "shared decision-mak*" or "shared decision mak*").ti,ab,kf.

Date limits 2000-2023

("improv* health" or improv* or well* or "community connect*" or benefit* or inclusion or "quality of life" or "quality-of-life").ti,ab,kf.

Date limits 2000-2023

(("social determinant*" or "social risk*" or "social need*") and (identifi* or screen* or referral* or address* or navigat*)).ti,ab,kf.

Date limits 2000-2023

("social prescribing" or "social prescription" or "patient navigat*" or "system navigat*" or "non-medical" or "non medical" or "social medicine" or "social participation" or "non-medical intervention" or well* or community or "link worker" or connect* or "care coordination" or " primary care" or "social refer*" or "social needs screening" or "social needs referral" or "care coord*").ti,ab,kf.

Date limits 2000-2023

| **Main Concept:** | ***Co-design or Co-production [outcome]*** | ***SP purpose for client [population]*** | ***Range of SP initiatives [intervention]*** | ***Social prescribing***  ***[intervention]*** |
| --- | --- | --- | --- | --- |
| **Search Terms:** | (codesign OR "co-design" OR coproduction OR "co-production" OR cocreat* OR "co-creat*" OR collaborat*OR engag* OR involve*OR codevelop* OR "co-develop*"OR "shared decision-mak*"OR "shared decisionmak*") | ("improv* health" OR improv* OR well* OR "community connect*" OR benefit* OR inclusion OR "quality of life" OR "quality-of-life") | (("social determinant*" OR "social risk*" OR "social need*") AND (identifi* OR screen* OR referral* OR address* OR navigat*)) | (“social prescribing” OR “social prescription” OR "patient navigat*" OR system navigat*" OR "non-medical" OR "non medical" OR "social medicine" OR "social participation" OR "non-medical intervention" OR well* OR community OR "link worker" connect* OR “care coordination” OR “primary care” OR “social refer*”) |
